# ^18^F-fluoromisonidazole predicts evofosfamide uptake in pancreatic tumor model

**DOI:** 10.1186/s13550-018-0409-1

**Published:** 2018-06-18

**Authors:** Milan Grkovski, Louise Fanchon, Naga Vara Kishore Pillarsetty, James Russell, John L. Humm

**Affiliations:** 1 Department of Medical Physics, Memorial Sloan Kettering Cancer Center, 1275 York Avenue, New York, NY 10065 USA; 2 Department of Radiology, Memorial Sloan Kettering Cancer Center, New York, NY USA

**Keywords:** Evofosfamide, ^18^F-fluoromisonidazole, Hypoxia, Pancreatic cancer, Personalized medicine

## Abstract

**Background:**

Quantitative imaging can facilitate patient stratification in clinical trials. The hypoxia-activated prodrug evofosfamide recently failed a phase III trial in pancreatic cancer. However, the study did not attempt to select for patients with hypoxic tumors. We tested the ability of ^18^F-fluoromisonidazole to predict evofosfamide uptake in an orthotopic xenograft model (BxPC3).

**Methods:**

Two forms of evofosfamide were used: (1) labeled on the active moiety (^3^H) and (2) on the hypoxia targeting nitroimidazole group (^14^C). Tumor uptake of evofosfamide and ^18^F-fluoromisonidazole was counted ex vivo. Autoradiography of ^14^C and ^18^F coupled with pimonidazole immunohistochemistry revealed the spatial distributions of prodrug, radiotracer, and hypoxia.

**Results:**

There was significant individual variation in ^18^F-fluoromisonidazole uptake, and a significant correlation between normalized ^18^F-fluoromisonidazole and both ^3^H-labeled and ^14^C-labeled evofosfamide. ^18^F-fluoromisonidazole and ^14^C-evofosfamide both localized in hypoxic regions as identified by pimonidazole.

**Conclusion:**

^18^F-fluoromisonidazole predicts evofosfamide uptake in a preclinical pancreatic tumor model.

## Introduction

Evofosfamide (TH302) is a hypoxia-activated prodrug that showed promise against pancreatic cancer in preclinical and phase II trials [1, 2]. The drug consists of a nitroimidazole moiety linked to a bromo-isophosphoramide mustard (Br-IPM). Enzymatic reduction in the absence of oxygen leads to the cleavage of evofosfamide. Neither the nitroimidazole nor the uncleaved evofosfamide molecule is significantly cytotoxic, but the liberated Br-IPM is diffusible and acts as a cytotoxic agent throughout the tumor. However, in a recent phase III MAESTRO study against pancreatic cancer, evofosfamide narrowly missed its primary endpoint [3]. The study design did not include hypoxia imaging at baseline, leading to the likely inclusion of patients with normoxic tumors that are not expected to benefit from such therapies. While pancreatic cancers are in general expected to be hypoxic [4], recent imaging studies have shown that not all pancreatic tumors display significant hypoxia [5–7].

In this study, we sought to determine whether ^18^F-fluoromisonidazole (^18^F-FMISO) could predict the tumor uptake of evofosfamide in a preclinical pancreatic tumor model, which if true would support the use of hypoxia imaging as way to identify likely drug responsive patients in the treatment population.

## Materials and methods

All animal experiments and procedures were approved by our Institutional Animal Care and Use Committee and complied with the National Institutes of Health regulations on the research use of rodents.

Six-week-old-female athymic mice were obtained from Envigo Laboratories (Indianapolis, IN) and maintained according to the Guide for the Care and Use of Laboratory Animals in an AAALAC-approved facility. BxPC3 cells (ATCC, Manassas, VA.) were implanted orthotopically in the pancreas. In preliminary experiments, we established the level of tumor hypoxia in this model through pimonidazole staining (Hypoxyprobe, Burlington, MA.). Six tumors were sectioned at four different depths/tumor. Pimonidazole was given at 100 mg/kg i.p. 1 h before sacrifice and stained as previously described [8].

**Fig. 1:**
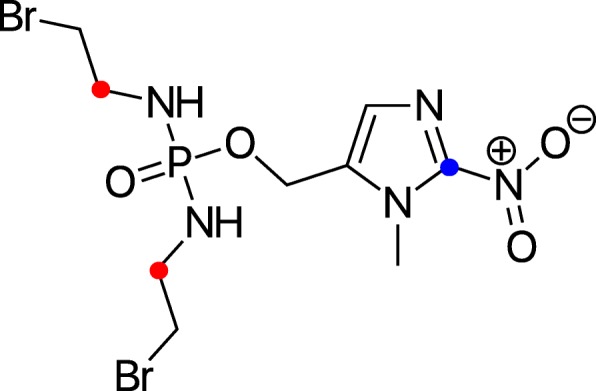
Radiolabeled evofosfamide. Drug was labeled either with tritium (red) or ^14^C (blue)

^18^F-fluoromisonidazole was synthesized on site [9] by the MSKCC Cyclotron and Radiochemistry Service. ^14^C-evofosfamide and ^3^H-evofosfamide were supplied by Merck pharmaceuticals (Germany). The ^14^C label is present on the inactive nitroimidazole ring; the ^3^H label on the bromo-isophosphoramide mustard (Fig. 1). All injections were intraperitoneal. Mice were co-injected with ^18^F-fluoromisonidazole and either ^3^H-evofosfamide and ^14^C-evofosfamide together (ex vivo counting; *n* = 20) or ^14^C-evofosfamide and pimonidazole (autoradiography; *n* = 3). For ex vivo counting, animals were injected with 3.7–5.6 MBq ^18^F, 740 kBq ^3^H, and 370 kBq ^14^C. For autoradiography, approximately 18.5 MBq ^18^F was injected per mouse along with 370 kBq ^14^C. Unlabeled evofosfamide (100 mg/kg) was co-injected with tracers.

Two hours after injection, mice were sacrificed and tumors removed. Tumors were either frozen and prepared for autoradiography and pimonidazole immunohistochemistry, or weighed, counted for ^18^F, and digested in Scintigest (Fisher Scientific, NJ)/hydrogen peroxide for counting on a liquid scintillation counter. Activity was expressed relative to sample weight and injected dose, and normalized to the tumor with the lowest ^14^C uptake.

^18^F and ^14^C images were obtained by a two-exposure technique. The first, which was obtained immediately post sacrifice, was for 2 h and provides a signal dominated by the ^18^F. The second (for 14 days) was captured after complete decay of the ^18^F and reveals the distribution of the ^14^C. Autoradiographic and immunohistochemical images were co-registered with fiduciary markers as previously described [10].

The correlation strength between indices was analyzed using Pearson’s *r*. For that purpose, autoradiography images were downsampled to 1 pixel = 0.1 mm. *p* < 0.05 was assumed to represent statistical significance.

## Results

### In vivo uptake of ^18^F-fluoromisonidazole and evofosfamide

**Fig. 2:**
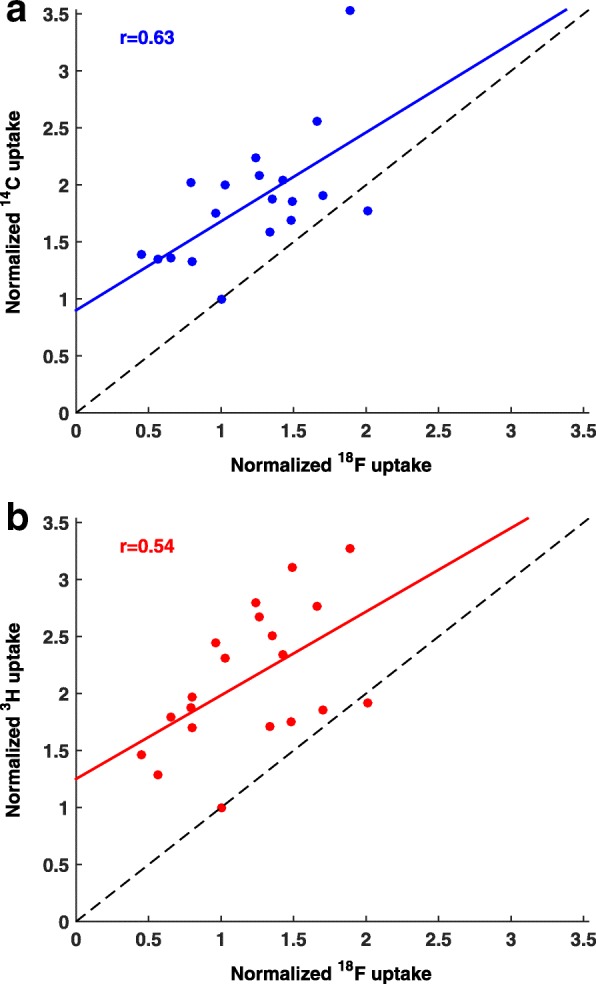
Whole-tumor correlation between ^18^F-fluoromisonidazole and ^3^H- or ^14^C-evofosfamide. Uptake of ^18^F-fluoromisonidazole versus ^14^C-evofosfamide (**a**) and ^3^H-evofosfamide (**b**). Data from 20 individual mice, normalized to the tumor with lowest evofosfamide uptake. The dashed line has a slope of 1, representing the ideal situation, where tracer and drug behave identically

In preliminary experiments, we found substantial inter-tumor variation in the pimonidazole positive fraction in the BxPC3 model (mean = 0.18, range = 0.09–0.30; *n* = 6) as quantified by Otsu thresholding (Each tumor was assessed at four depths, separated by 1 mm. Necrotic tissue was excluded based on hematoxylin and eosin staining.). Based on this, we co-administered ^18^F-fluoromisonidazole, ^14^C-evofosfamide, ^3^H-evofosfamide, and unlabeled evofosfamide in a therapy dose to 20 tumor-bearing mice. Observed inter-tumor variation in normalized ^18^F-fluoromisonidazole counts correlated significantly with both ^14^C-evofosfamide and ^3^H-evofosfamide uptake (*r* = 0.63 and 0.54, respectively, *p* ≤ 0.01; Fig. 2). The correlation between ^14^C-evofosfamide and ^3^H-evofosfamide was 0.81 (*p* < 0.01).

### Co-localization of ^18^F-fluoromisonidazole and evofosfamide

**Fig. 3:**
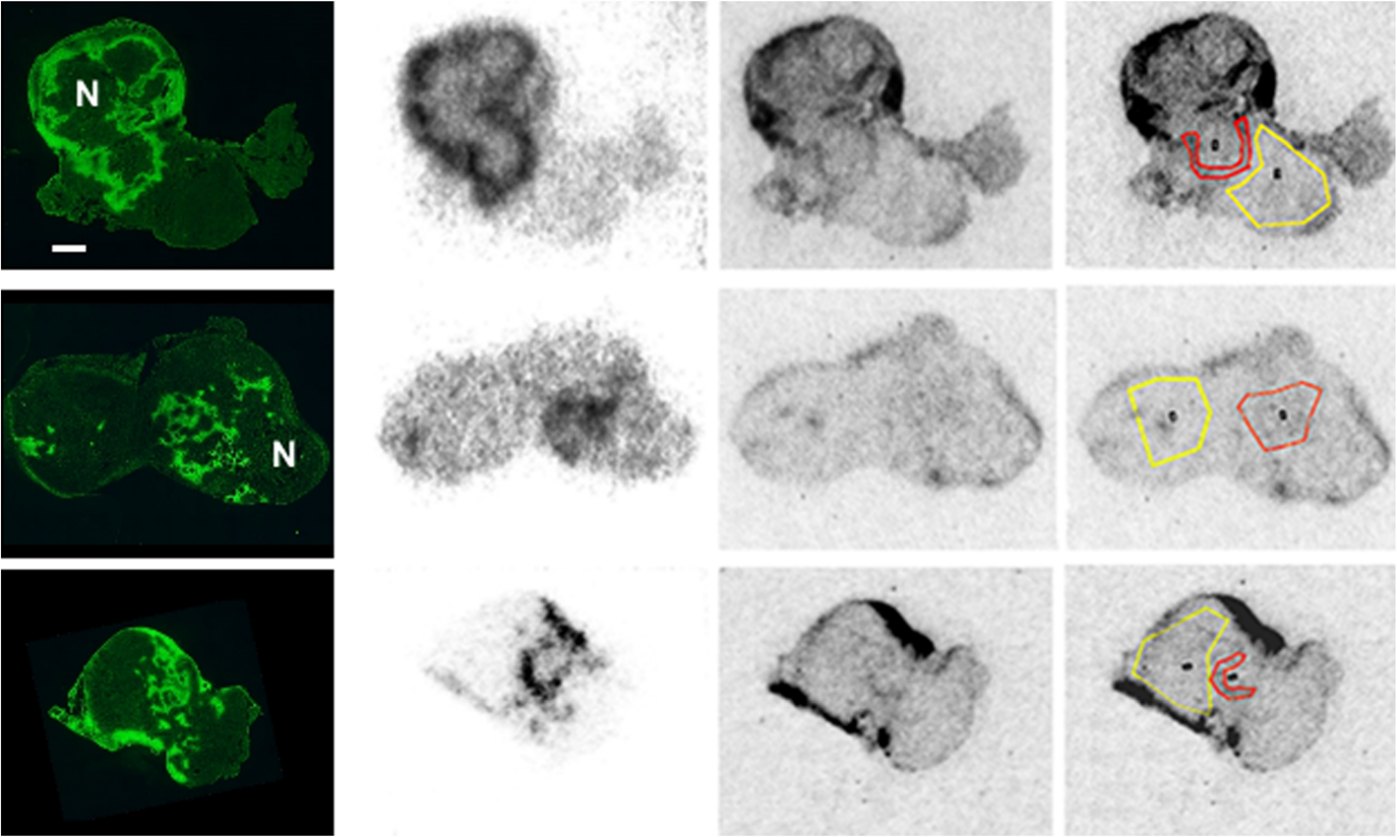
Microscopic correlation between ^18^F-fluoromisonidazole and ^14^C-evofosfamide. Each row contains matched images from one of three individual tumors. Columns from left to right: pimonidazole, ^18^F-fluoromisonidazole, ^14^C-evofosfamide, and ^14^C evofosfamide image with ROIs for quantitation superimposed. “Hotspot” outlined in red; background in yellow. Necrosis (N) was identified on adjacent sections stained by hematoxylin and eosin. Scale bar = 1 mm

Digital autoradiography helped clarify the scatter plots. ^18^F-fluoromisonidazole and ^14^C-evofosfamide both preferentially accumulated in hypoxic regions as identified by immunofluorescence staining for pimonidazole (Fig. 3; the higher resolution of the ^14^C image is due to the lower energy and consequent shorter path length of ^14^C β particles). Pearson’s correlation between downsampled autoradiography images showing uptake patterns of ^18^F-fluoromisonidazole and ^14^C-evofosfamide was 0.62, 0.19, and 0.59 for the three tumor sections, respectively. Activity in ^14^C hotspots was 52% greater than the bulk of the viable tissue. In the matched regions of the ^18^F images, activity was 4.4 times greater than adjacent tissue.

## Discussion

The objective of this study was to examine whether ^18^F-fluoromisonidazole could predict the tumor uptake of evofosfamide in a preclinical pancreatic tumor model. Our findings confirmed a linear correlation between ^18^F-fluoromisonidazole over the concentration range studied, supporting the notion that ^18^F-fluoromisonidazole could stratify a patient population according to those most likely to benefit from evofosfamide therapy. In all investigated tumors, ^18^F-fluoromisonidazole localized in the hypoxic regions as identified by pimonidazole staining, and digital autoradiography revealed co-localization between ^18^F-fluoromisonidazole and ^14^C-evofosfamide. The dynamic range of the ^14^C-evofosfamide signal was lower than ^18^F-fluoromisonidazole, a possible consequence of saturation of the reductases at therapy-associated concentrations of the drug. However, in mice, multiple low doses of evofosfamide were not more effective than single high doses [1]. The reduced sensitivity of evofosfamide uptake to hypoxia could account for the regression slope being less than one and might relate to the large dose of drug saturating the enzyme reduction system. This is supported by the fact that the spatial match between evofosfamide and pimonidazole was much closer when only tracer doses of evofosfamide were given (data not shown).

The status of evofosfamide is now in considerable doubt given the outcome of the recent MAESTRO trial, which indicated a modest benefit for evofosfamide combined with gemcitabine compared with gemcitabine alone in patients with unresectable locally advanced or metastatic pancreatic ductal adenocarcinoma, causing the sponsoring company to abandon the drug. Based on the reported range of tumor hypoxia in patients with pancreatic cancer [6], it is highly likely that the trial population would have included non-hypoxic tumors and that these individuals would potentially have masked the benefit of evofosfamide. Such an effect was shown with tirapazamine, an earlier hypoxia-activated prodrug, where a retrospective analysis demonstrated a significant advantage for the drug in head and neck disease, but only for patients with hypoxic tumors as assessed by ^18^F-fluoromisonidazole PET [11]. Our results suggest that ^18^F-fluoromisonidazole could have provided similar information for the evofosfamide trial.

## Conclusion

Uptake and distribution of ^18^F-fluoromisonidazole and evofosfamide correlate in vivo on both whole-tumor and microscopic levels, indicating a potential for the use of ^18^F-fluoromisonidazole as a quantitative biomarker of evofosfamide uptake in pancreatic tumors.
